# Association of novel obesity-related lipid markers with diabetes prevalence in high-risk middle-aged and older adults with cardiovascular diseases

**DOI:** 10.3389/fpubh.2025.1737669

**Published:** 2026-01-26

**Authors:** Mingsi Chen, Xue Zhou, Keying Yang, Qiaojun Li, Liying Zhao, Xingren Wang, Ying Lu

**Affiliations:** 1 Department of Environmental and Occupational Health, School of Public Health, Hainan Medical University, Haikou, China; 2 School of Public Health, Key Laboratory of Tropical Translational Medicine of Ministry of Education, Hainan Medical University, Haikou, China; 3 Hainan Center for Disease Control and Prevention, Haikou, China; 4 Hainan Academy of Preventive Medicine, Haikou, China

**Keywords:** diabetes, lipid indicators, middle-aged and older adults, obesity indicators, people at high risk of cardiovascular disease

## Abstract

**Objective:**

This study investigated the association between obesity and lipid markers including the Chinese Visceral Adiposity Index (CVAI), triglyceride-glucose waist-to-height ratio (TyG-WHtR), triglyceride-glucose waist circumference (TyG-WC), triglyceride-glucose body mass index (BMI; TyG-BMI), and conicity index (C-index) with the presence of diabetes among individuals at high cardiovascular disease (CVD) risk in Hainan Province, China, and providing evidence to support early identification of individuals with elevated metabolic burden.

**Methods:**

Between February 2023 and January 2024, a multistage stratified cluster sampling methodology was used for an initial screening of 6,148 individuals across eight cities and counties in Hainan Province. This screening involved physical measurements, face-to-face questionnaires, and laboratory tests, ultimately identifying 1,603 individuals at high risk for cardiovascular disease. For these high-risk individuals, various indicators were calculated based on their lipid profiles, waist circumference, height, and weight, including the CVAI, TyG-WHtR, TyG-WC, TyG-BMI, and the C-index. Logistic regression and restricted cubic spline (RCS) models were used to evaluate the associations and linear and non-linear associations between these indicators and the presence of diabetes. Additionally, receiver operating characteristic (ROC) curves were generated to evaluate the discriminatory ability of these indicators in distinguishing individuals with and without diabetes within the high-risk cardiovascular disease populations.

**Results:**

This study included 1,603 individuals at high risk for cardiovascular disease, of whom 330 (20.59%) had diabetes. After adjusting for confounding factors using logistic regression, the odds of having diabetes were higher in the Q4 group than in the Q1 group for the CVAI, TyG-WHtR, TyG-WC, and TyG-BMI indicators, but not for the C-index. The corresponding odds ratios (OR) and 95% confidence intervals (CI) were as follows: CVAI was 2.43 (95% CI: 1.41–4.17), TyG-WHtR was 12.80 (95% CI: 7.46–21.96), TyG-WC was 16.41 (95% CI: 9.21–29.24), and TyG-BMI was 5.28 (95% CI: 3.23–8.64). The RCS analyses showed that CVAI, TyG-WHtR, TyG-BMI, and the C-index were positively linearly associated with the presence of diabetes, whereas TyG-WC exhibited a positive nonlinear association with diabetes presence (P < 0.05). ROC curve analysis indicated that TyG-WC had the strongest discriminatory ability, with an area under the curve (AUC) of 0.735 (95% CI: 0.705–0.766), followed by the TyG-WHtR index, which had an AUC of 0.716 (95% CI: 0.685–0.747). The optimal cutoff values determined based on the maximum Youden index were TyG-WC at 797.89 and TyG-WHtR at 5.08, respectively.

**Conclusion:**

CVAI, TyG-WHtR, TyG-WC, TyG-BMI, and the C-index were positively associated with diabetes in middle-aged and older adults at high cardiovascular risk. CVAI, TyG-WHtR, TyG-WC, and TyG-BMI showed good discriminatory performance for identifying diabetes, with TyG-WC performing best. These TyG-based and obesity-related composite indicators provide feasible options for diabetes identification and risk stratification in high cardiovascular-risk populations and may support population-level screening and prevention efforts in aging societies.

## 1 Introduction

Diabetes mellitus (DM) represents a major global public health challenge, contributing to substantial healthcare and socioeconomic burdens worldwide. This burden is further amplified in the context of population aging, as middle-aged and older adults account for a large proportion of individuals affected by diabetes and its complications. Moreover, DM is a critical risk factor for cardiovascular disease (CVD) and all-cause mortality. In China, more than 118 million individuals are affected by diabetes, accounting for ~22% of the global diabetic population (1). Notably, the prevalence of diabetes increases markedly with age, with the prevalence of CVD being significantly higher among individuals with diabetes compared to non-diabetic populations (2); Furthermore, the prevalence of CVD among individuals with type 2 diabetes ranges from 29.4 to 46.0%, which is approximately two to four times higher than that observed in non-diabetic populations (3). Among middle-aged and older adults, prolonged exposure to metabolic abnormalities and the accumulation of multiple cardiometabolic risk factors further exacerbate cardiovascular risk. Given the escalating burden of diabetes and its complications, particularly in middle-aged and older populations, early identification of individuals with elevated metabolic risk, even before the development of overt hyperglycemia is of great importance. Although fasting plasma glucose (FPG) and glycated hemoglobin (HbA1c) are well-established diagnostic standards for diabetes, they may be less sensitive to early or subclinical metabolic alterations (4). This limitation is especially relevant for middle-aged and older adults, in whom metabolic dysregulation often develops gradually and may remain undetected for extended periods. Thus, simple and integrative metabolic indicators that complement traditional biomarkers may offer additional insights for early metabolic risk assessment, thereby supporting earlier public health intervention and prevention strategies in aging populations.

In recent years, the triglyceride-glucose (TyG) index and its derived indicators, such as TyG-BMI, TyG-WC, and TyG-WHtR, have gained increasing attention (5, 6). Accumulating evidence suggests that these indicators are strongly associated with insulin resistance, dyslipidemia, and early metabolic dysfunction. Importantly, these composite indicators may reflect both lipid–glucose metabolism and central obesity, offering an integrated view of metabolic disturbances associated with the presence of diabetes, rather than directly implying causation (7, 8). However, evidence remains limited for middle-aged and older adults at high cardiovascular risk, who typically present with more pronounced metabolic abnormalities and therefore require more precise early risk identification.

To provide additional evidence in this field, this study investigates the associations between diabetes and multiple metabolic indicators—including TyG-derived indices (TyG-BMI, TyG-WC, TyG-WHtR) and adiposity-related markers (CVAI and C-index)—in a large population at high cardiovascular risk in Hainan Province, China. By focusing on middle-aged and older adults, this study aims to provide evidence of public health relevance for diabetes prevention and metabolic risk stratification in aging societies. Rather than replacing established diagnostic criteria such as fasting plasma glucose (FPG) or glycated hemoglobin (HbA1c), this study evaluates whether these indicators may help identify individuals more likely to present with diabetes-related metabolic abnormalities, thereby supporting more individualized clinical assessment.

## 2 Objects and methods

### 2.1 Research subjects

**Figure 1:**
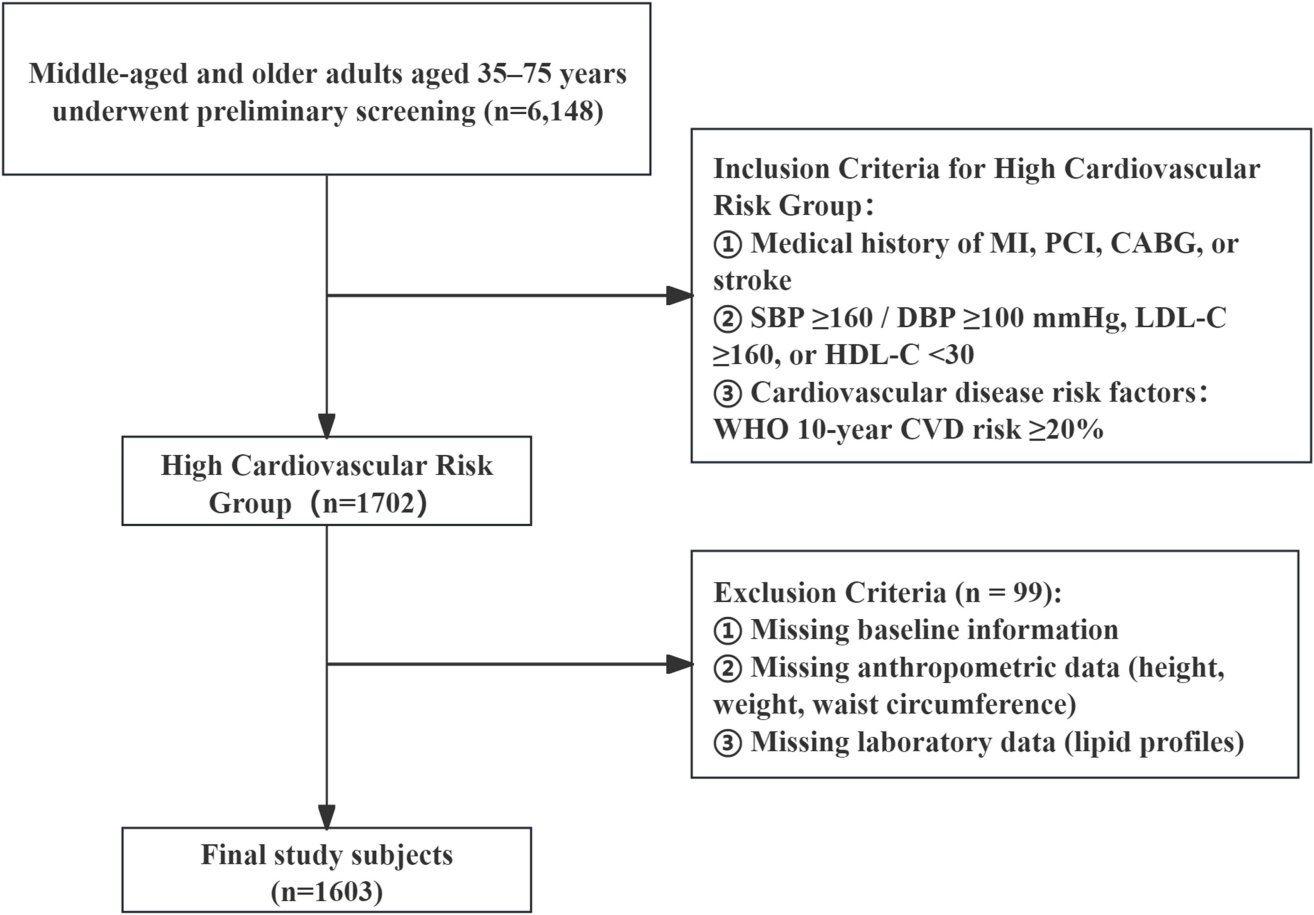
Flowchart of participant selection for the cardiovascular high-risk population.

From February 2023 to January 2024, this study employed a multi-stage stratified cluster sampling method to select eight counties and cities in Hainan Province as survey areas. The selection of these sites was based on factors including regional representativeness, urban-rural population distribution, ethnic composition, and operational feasibility. In addition, according to the provincial urban-to-rural permanent population ratio of 1:3, Xiuying District of Haikou City in the eastern part of Hainan and Danzhou City in the western part of Hainan were designated as urban survey sites, while the remaining areas—Wenchang City, Qionghai City, and Wanning City in eastern Hainan, as well as Sanya City, Ledong County, and Dongfang City in western Hainan—were designated as rural survey sites. The study population comprised permanent residents aged 35–75 years who had lived in the region for at least 6 months. A total of 6,148 individuals underwent preliminary screening. Participants were classified as high-risk for cardiovascular disease if they met any one of the following criteria (9): ① Medical history (meeting any one of the following four conditions): (a) History of myocardial infarction; (b) History of percutaneous coronary intervention (PCI); (c) History of coronary artery bypass grafting (CABG); (d) History of stroke (ischemic or hemorrhagic). ② Blood pressure and lipid levels (meeting any of the following three conditions): (a) Systolic blood pressure (SBP) ≥160 mmHg or diastolic blood pressure (DBP) ≥100 mmHg; (b) Low-density lipoprotein cholesterol (LDL-C) ≥160 mg/dl (4.14 mmol/L); (c) High-density lipoprotein cholesterol (HDL-C) <30 mg/dl (0.78 mmol/L). ③ Cardiovascular disease risk factors: Cardiovascular disease risk assessment was conducted for all screened individuals using the risk assessment prediction chart from the 2008 World Health Organization Guidelines for the Assessment and Management of Cardiovascular Risk (10), which includes assessment indicators such as age, gender, systolic blood pressure (SBP), smoking status, history of diabetes, and hypercholesterolemia. If the predicted 10-year risk of cardiovascular disease was ≥20%, the individual was classified as high risk. After screening, 1,702 individuals met the inclusion criteria. Following the exclusion of 99 participants due to missing baseline data, anthropometric measurements (height, weight, waist circumference), or laboratory results (lipid profiles), a total of 1,603 participants were included in the final analysis (Figure 1). This study was approved by the Ethics Committee of Fuwai Hospital, Chinese Academy of Medical Sciences [Approval No. (2014) 574], and written informed consent was obtained from all participants. (1) identifying the institutional and/or licensing committee approving the experiments, including any relevant details; (2) confirming that all experiments were performed in accordance with relevant guidelines and regulations.

### 2.2 Methods

#### 2.2.1 Survey questionnaire

(1) The questionnaire was administered by professionally trained personnel through face-to-face and telephone interviews, covering demographic characteristics (e.g., gender, age, education level, urban/rural residence, annual household income), lifestyle behaviors (e.g., smoking and alcohol consumption, physical activity, dietary pattern), and medical history (e.g., hypertension and other chronic diseases, family history of diabetes). (2) Physical examination: Physical examinations were conducted in a quiet, standardized environment. Participants were asked to stand upright with their chest lifted and head raised, remove their shoes and hats, and wear light clothing. Height and weight were automatically measured using an ultrasonic stadiometer, and waist circumference was measured horizontally. All measurements followed a unified protocol with standardized instruments and methods to ensure data consistency and quality control.

#### 2.2.2 Laboratory tests

Fasting blood glucose and lipid profiles were measured under standardized conditions using validated portable analyzers. The lipid profile included total cholesterol (TC), triglycerides (TG), high-density lipoprotein cholesterol (HDL-C), and low-density lipoprotein cholesterol (LDL-C).

#### 2.2.3 Diagnostic criteria

Diabetes was diagnosed according to the following criteria: (1) fasting blood glucose (FBG) ≥ 7.0 mmol/L after ≥8 h of fasting, or FBG ≥11.1 mmol/L when fasting duration was <8 h or unknown; (2) self-reported use of blood glucose-lowering medications (specify the medication name or indicate if the name is unknown); (3) self-reported history of diabetes. Dyslipidemia is defined as meeting any of the following criteria (11): dyslipidemia: TC > 6.2 mmol/L or TG > 2.3 mmol/L or LDL-C > 4.1 mmol/L or HDL-C <1.0 mmol/L; central obesity is defined (12) as a waist circumference ≥ 90 cm for men and ≥ 85 cm for women.

#### 2.2.4 Calculation of obesity and lipid-related indicators

① BMI = weight (kg)/height^2^ (m^2^); ② WHtR = waist circumference (m)/height (m); ③ CVAI (male) = −267.93 + 0.68 × age + 0.03 × BMI + 4.00 × WC + 22.00 × Log_10_TG × 16.32 × HDL-C; ④ CVAI (female) = −187.32 + 1.71 × age + 4.23 × BMI + 1.12 × WC + 39.76 × Log_10_TG−11.66 × HDL-C; ⑤ C-index = 0.109–1 × WC (m) × [Weight (kg)/Height (m)]^−1/2^; ⑥ TyG index = ln[TG (mg/dl) × FBG (mg/dl)/2]; ⑦ TyG-WHtR = TyG × WHtR; ⑧ TyG-WC = TyG × WC; ⑨ TyG-BMI = TyG × BMI. TG and HDL-C were measured in mmol/L (13–15).

### 2.3 Statistical methods

Statistical analyses were performed using SPSS version 27.0 and R version 4.0.3. Continuous variables with a normal distribution were expressed as mean ± standard deviation and compared using independent-sample *t*-tests. Non-normally distributed variables were expressed as median (interquartile range) [M (P25, P75)] and compared using the Mann–Whitney *U* test. Categorical variables were presented as frequencies (percentages) and compared using the chi-squared test. Participants were divided into quartiles (Q1–Q4) according to the distribution of CVAI, C-index, and TyG-derived indicators. Multivariable logistic regression models were used to assess the associations between each metabolic index and the presence of diabetes, with OR and 95% CI reported. RCS models were applied to explore potential nonlinear associations between metabolic indicators and the presence of diabetes. ROC curve analysis was conducted to evaluate the discriminatory ability of CVAI, C-index, and TyG-derived indicators. The optimal cutoff values were identified based on the maximum Youden index, and the DeLong test was used to compare AUCs. Subgroup analyses were performed for variables significantly associated with diabetes in the multivariable models, stratified by age (<60 vs. ≥60 years), sex (male vs. female), smoking status (yes vs. no), and central obesity (yes vs. no). A two-sided *P*-value <0.05 was considered statistically significant.

The selection of covariates was guided by the results of univariate analyses as well as by established associations with metabolic indicators and diabetes reported in previous epidemiological studies and clinical practice. All variables that were statistically significant in the univariate analysis—including age, sex, urban or rural residence, dyslipidemia, central obesity, smoking status, hypertension, medication history, physical activity, and family history of diabetes—were included in the adjusted model. Although drinking history did not reach statistical significance in the univariate analysis, it was retained because alcohol consumption is known to influence lipid metabolism and insulin resistance and may therefore act as a potential confounder. To ensure appropriate model adjustment and to minimize the risk of under or over-adjustment, variables identified in the literature as potential confounders were preserved even if they were not statistically significant in the univariate analyses.

### 2.4 Quality control

Quality control measures encompassed protocol development, staff training, equipment preparation, site setup, standardized examination procedures, and data management. Before project implementation, the Disease Control Bureau of the National Health and Family Planning Commission and the National Center for Cardiovascular Diseases organized expert panels to revise technical protocols to ensure scientific rigor and feasibility. The National Center for Cardiovascular Diseases was responsible for training medical staff, evaluating their proficiency, issuing certifications, and organizing regular retraining. During the study, quality control procedures were applied to all stages, including physical examination and sample collection. Field inspections and central data monitoring were conducted to standardize operations and resolve issues promptly. Participating centers were required to upload data in a timely manner for national and provincial monitoring. To ensure laboratory accuracy, blood and urine samples were sent to the National Center for Cardiovascular Diseases for validation.

## 3 Results

Basic information of the cardiovascular high-risk population among the 1,603 high-risk individuals included in the study, 714 (44.54%) were men and 889 (55.46%) were women, with an average age of (60.01 ± 9.43) years. The number of individuals with diabetes was 330, with a prevalence rate of 20.59%. Significant between-group differences were observed in sex, urban–rural distribution, smoking status, physical activity, hypertension, dyslipidemia, medication use, and family history of diabetes (*P* < 0.05). Patients with diabetes exhibited significantly higher BMI, WC, WHtR, CVAI, C-index, TyG, TyG-WHtR, TyG-WC, and TyG-BMI compared with those without diabetes (*P* < 0.05). In contrast, no significant differences were found in educational level, annual income, healthy dietary pattern, or alcohol consumption between diabetic and non-diabetic individuals (*P* > 0.05) Tables 1, 2.

**Table 1:** Baseline characteristics of study participants with and without diabetes in the cardiovascular high-risk population.

**Variables**	**Total (*n* = 1,603)**	**Non-DM (*n* = 1,273)**	**DM(*n* = 330)**	***χ^2^***	***P*-value**
**Sex [*****n*** **(%)]**					
Male	714 (44.54)	536 (42.11)	178 (53.94)	14.86	<0.001
Female	889 (55.46)	737 (57.89)	152 (46.06)		
Age (year), mean (SD)	60.01 ± 9.43	59.71 ± 9.60	61.21 ± 8.65	−2.74	0.006
**Education [*****n*** **(%)]**					
Junior high school and below	1,319 (82.28)	1,046 (82.17)	273 (82.73)	0.10	0.950
High school/vocational school	232 (14.47)	186 (14.61)	46 (13.94)		
College graduate or above	52 (3.24)	41 (3.22)	11 (3.33)		
**Urban-rural divide [*****n*** **(%)]**					
Rural	1,074 (67.00)	869 (68.26)	205 (62.12)	4.47	0.034
Urban	529 (33.00)	404 (31.74)	125 (37.88)		
**Dyslipidemia [*****n*** **(%)]**					
No	1,110 (69.25)	903 (70.93)	207 (62.73)	8.29	0.004
Yes	493 (30.75)	370 (29.07)	123 (37.27)		
**Central obesity [*****n*** **(%)]**					
No	1,001 (62.45)	834 (65.51)	167 (50.61)	24.84	<0.001
Yes	602 (37.55)	439 (34.49)	163 (49.39)		
**Annual income [*****n*** **(%), million yuan]**					
<5 million yuan	1,265 (78.91)	1,013 (79.58)	252 (76.36)	1.63	0.202
≥5 million yuan	338 (21.09)	260 (20.42)	78 (23.64)		
**Drinking [*****n*** **(%)]**					
No	1,490 (92.95)	1,186 (93.17)	304 (92.12)	0.44	0.509
Yes	113 (7.05)	87 (6.83)	26 (7.88)		
**Smoking [*****n*** **(%)]**					
No	1,248 (77.85)	1,008 (79.18)	240 (72.73)	6.33	0.012
Yes	355 (22.15)	265 (20.82)	90 (27.27)		
**Hypertension [*****n*** **(%)]**					
No	1,226 (76.48)	1,004 (78.87)	222 (67.27)	19.59	<0.001
Yes	377 (23.52)	269 (21.13)	108 (32.73)		
**Medication history [*****n*** **(%)]**					
No	1,206 (75.23)	1,030 (80.91)	176 (53.33)	106.97	<0.001
Yes	397 (24.77)	243 (19.09)	154 (46.67)		
**Physical activity [*****n*** **(%)]**					
No	422 (26.33)	356 (27.97)	66 (20.00)	8.57	0.003
Yes	1,181 (73.67)	917 (72.03)	264 (80.00)		
**Healthy dietary pattern [*****n*** **(%)]**					
No	611 (38.12)	473 (37.16)	138 (41.82)	2.41	0.120
Yes	992 (61.88)	800 (62.84)	192 (58.18)		
**Family history of diabetes [*****n*** **(%)]**					
No	1,571 (98.00)	1,255 (98.59)	316 (95.76)	10.72	0.001
Yes	32 (2.00)	18 (1.41)	14 (4.24)		

**Table 2:** Obesity- and lipid-related indicators in cardiovascular high-risk participants with and without diabetes.

**Variables, mean (SD)**	**Total (*n* = 1,603)**	**Non-DM (*n* = 1,273)**	**DM (*n* = 330)**	***t***	***P*-value**
BMI, kg/m^2^	24.21 ± 3.24	24.02 ± 3.19	24.93 ± 3.37	−4.60	<0.001
WC, cm	84.39 ± 8.83	83.61 ± 8.59	87.40 ± 9.10	−7.06	<0.001
WHtR	0.53 ± 0.06	0.53 ± 0.05	0.55 ± 0.06	−4.67	<0.001
CVAI	99.09 ± 34.96	95.41 ± 34.33	113.28 ± 33.76	−8.46	<0.001
C-index	1.25 ± 0.08	1.25 ± 0.08	1.27 ± 0.08	−4.57	<0.001
TyG	7.34 ± 0.57	7.21 ± 0.47	7.86 ± 0.61	−18.04	<0.001
TyG-WHtR	4.78 ± 0.62	4.68 ± 0.58	5.17 ± 0.63	−13.49	<0.001
TyG-WC	755.59 ± 102.05	737.08 ± 93.03	826.97 ± 103.97	−14.29	<0.001
TyG-BMI	216.77 ± 34.65	211.81 ± 32.51	235.90 ± 36.03	−11.73	<0.001

Data are presented as mean (standard deviation).

BMI, body mass index; WC, waist circumference; WHtR, waist-to-height ratio; CVAI, Chinese Visceral Adiposity Index; TyG, triglyceride–glucose index.

Association between novel obesity-lipid indicators and diabetes in the unadjusted model, the odds of having diabetes were significantly higher in the Q4 group compared with Q1 for CVAI, TyG-WHtR, TyG-WC, TyG-BMI, and the C-index, with corresponding ORs (95% CIs) of 3.57 (2.47–5.15), 8.09 (5.33–12.28), 9.31 (6.04–14.35), 4.95 (3.37–7.27), and 2.24 (1.57–3.21), respectively. After adjusting for potential confounders (sex, urban–rural status, dyslipidemia, central obesity, smoking, hypertension, and medication history), the associations remained significant for CVAI, TyG-WHtR, TyG-WC, and TyG-BMI, with adjusted ORs (95% CIs) of 2.55 (1.49–4.34), 12.85 (7.52–21.96), 16.68 (9.41–29.58), and 5.01 (3.12–8.05), respectively. The association for the C-index was not statistically significant. In Model 3, which further adjusted for physical activity and family history of diabetes, the associations remained largely consistent. The corresponding ORs (95% CIs) for Q4 vs. Q1 were 2.43 (1.41–4.17) for CVAI, 12.80 (7.46–21.96) for TyG-WHtR, 16.41 (9.21–29.24) for TyG-WC, and 5.28 (3.23–8.64) for TyG-BMI, while the C-index remained non-significant Table 3.

**Table 3:** Logistic regression analysis of obesity- and lipid-related indicators associated with diabetes in a cardiovascular high-risk population.

**Indicators**	**Model 1**		**Model 2**		**Model 3**	
	***OR (95% CI)***	***P*** **-value**	***OR (95% CI)***	***P*** **-value**	***OR (95% CI)***	***P*** **-value**
**CVAI**						
Q1	1.00 (Reference)		1.00 (Reference)		1.00 (Reference)	
Q2	1.43 (0.95–2.13)	0.083	1.26 (0.82–1.93)	0.299	1.22 (0.79–1.87)	0.376
Q3	2.01 (1.37–2.95)	<0.001	1.63 (1.04–2.56)	0.034	1.58 (1.01–2.49)	0.050
Q4	3.57 (2.47–5.15)	<0.001	2.55 (1.49–4.34)	<0.001	2.43 (1.41–4.17)	0.001
**TyG-WHtR**						
Q1	1.00 (Reference)		1.00 (Reference)		1.00 (Reference)	
Q2	1.90 (1.20–3.03)	0.007	1.91 (1.18–3.11)	0.009	1.93 (1.19–3.14)	0.008
Q3	3.07 (1.98–4.76)	<0.001	3.54 (2.19–5.74)	<0.001	3.54 (2.18–5.74)	<0.001
Q4	8.09 (5.33–12.28)	<0.001	12.85 (7.52–21.96)	<0.001	12.80 (7.46–21.96)	<0.001
**TyG-WC**						
Q1	1.00 (Reference)		1.00 (Reference)		1.00 (Reference)	
Q2	2.12 (1.32–3.43)	0.002	2.10 (1.28–3.46)	0.003	2.12 (1.29–3.48)	0.003
Q3	3.42 (2.17–5.39)	<0.001	4.33 (2.61–7.17)	<0.001	4.29 (2.58–7.12)	<0.001
Q4	9.31 (6.04–14.35)	<0.001	16.68 (9.41–29.58)	<0.001	16.41 (9.21–29.24)	<0.001
**TyG-BMI**						
Q1	1.00 (Reference)		1.00 (Reference)		1.00 (Reference)	
Q2	1.44 (0.93–2.22)	0.100	1.41 (0.89–2.22)	0.139	1.42 (0.90–2.24)	0.127
Q3	2.73 (1.83–4.07)	<0.001	2.73 (1.75–4.24)	<0.001	2.83 (1.81–4.45)	<0.001
Q4	4.95 (3.37–7.27)	<0.001	5.01 (3.12–8.05)	<0.001	5.28 (3.23–8.64)	<0.001
**C-index**						
Q1	1.00 (Reference)		1.00 (Reference)		1.00 (Reference)	
Q2	1.42 (0.97–2.07)	0.068	1.09 (0.73–1.64)	0.664	1.11 (0.74–1.67)	0.613
Q3	1.83 (1.27–2.64)	0.001	1.16 (0.75–1.77)	0.509	1.17 (0.76–1.79)	0.483
Q4	2.24 (1.57–3.21)	<0.001	1.20 (0.75–1.92)	0.443	1.25 (0.78–2.01)	0.344

Model 1: unadjusted. Model 2: adjusted for age, sex, urban–rural residence, central obesity, dyslipidemia, drinking, smoking, hypertension, and medication history. Model 3: further adjusted for physical activity and family history of diabetes on the basis of Model 2.

**Figure 2:**
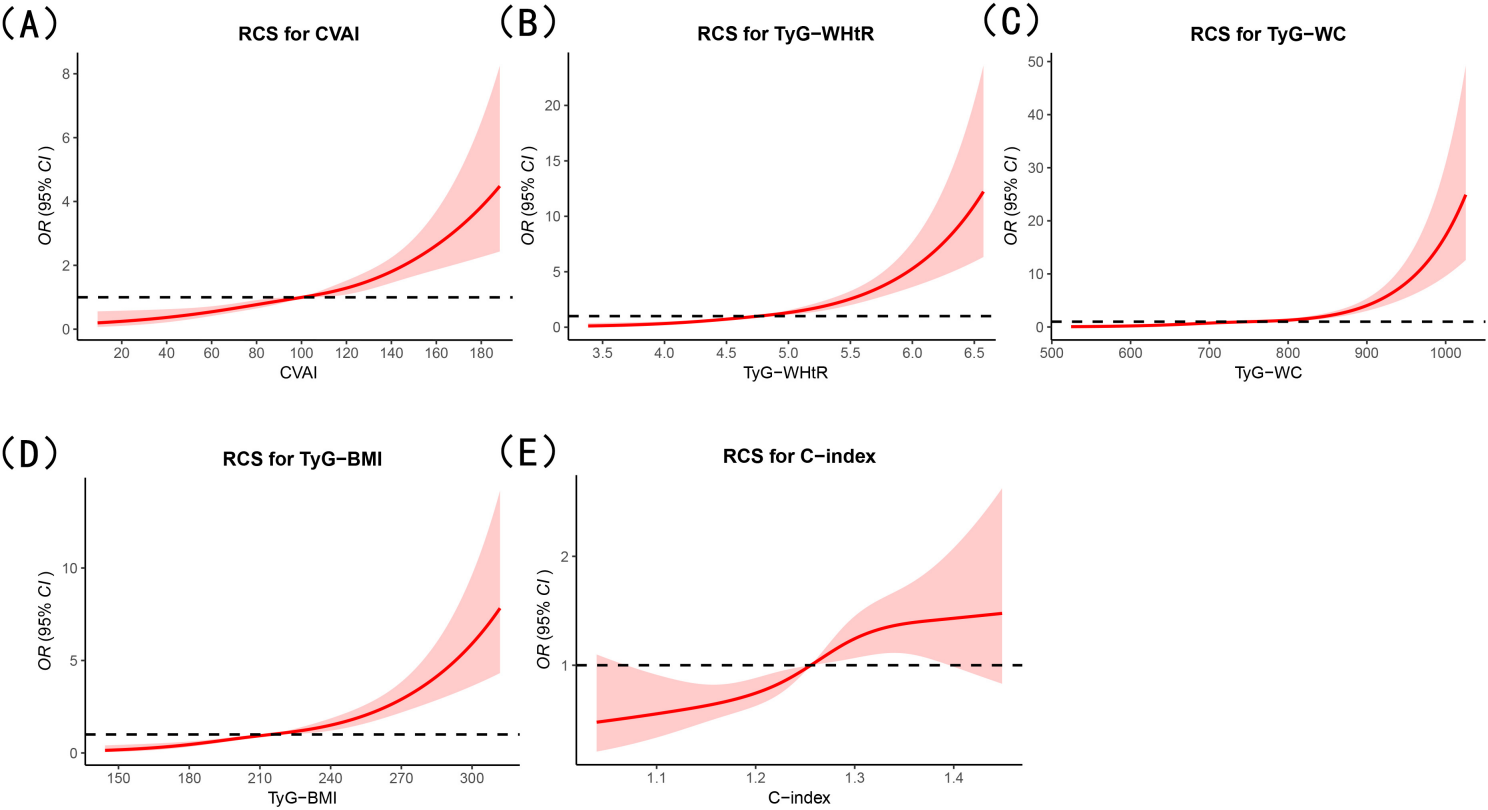
RCS analysis of the associations between obesity- and lipid-related indicators and diabetes prevalence in cardiovascular high-risk individuals. **(A)** Chinese Visceral Adiposity Index (CVAI); **(B)** triglyceride-glucose index-waist-to-height ratio (TyG-WHtR); **(C)** triglyceride-glucose index-waist circumference (TyG-WC); **(D)** triglyceride-glucose index-body mass index (TyG-BMI); **(E)** conicity index (C-index).

RCS analysis demonstrated significant positive linear associations between CVAI, TyG-WHtR, TyG-BMI, and the C-index with the presence of diabetes (*P* < 0.001; *P*-non-linearity > 0.05), while TyG-WC showed a significant nonlinear association with the presence of diabetes (*P* < 0.001; *P*-non-linearity <0.05). As CVAI, TyG-WHtR, TyG-WC, TyG-BMI, and C-index increased, the odds of having diabetes were higher among individuals with high cardiovascular risk (Figure 2).

**Figure 3:**
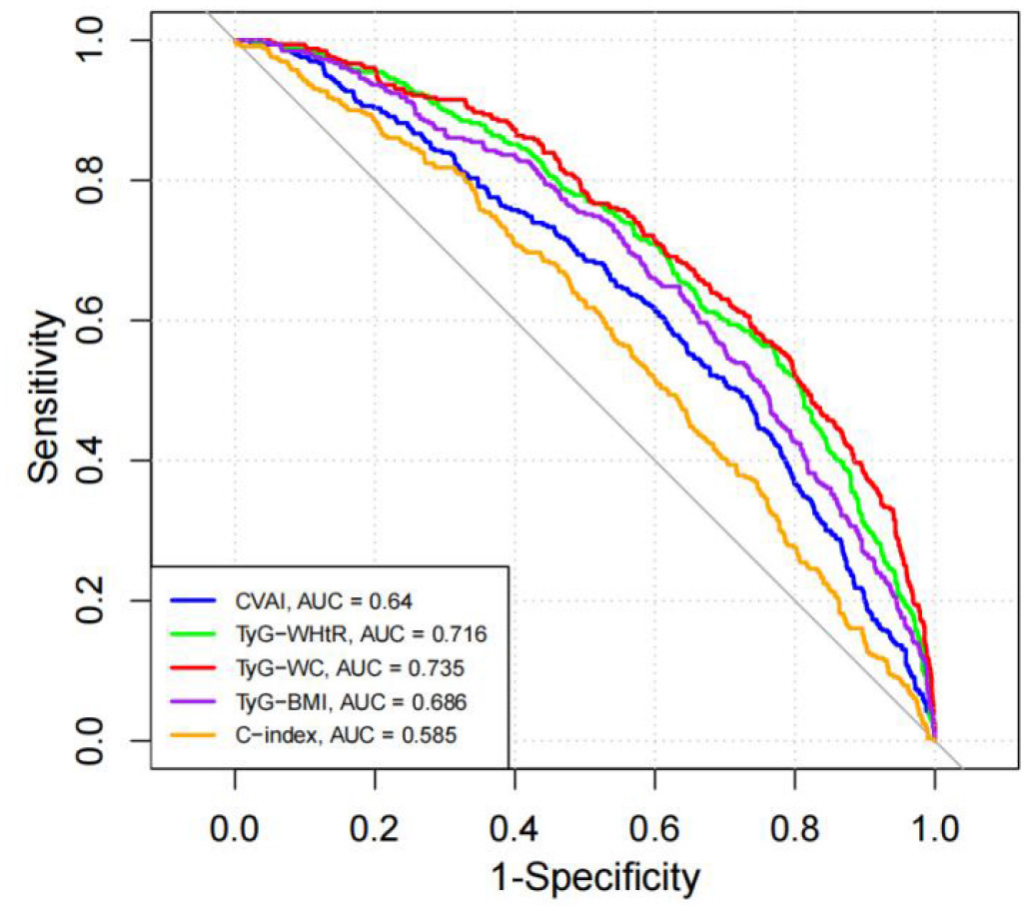
ROC curve analysis of obesity- and lipid-related indicators for identifying diabetes in a high cardiovascular-risk population.

ROC curve analysis of novel obesity-lipid indicators and the presence of diabetes in high-risk cardiovascular populations all obesity-lipid indicators (CVAI, TyG-WHtR, TyG-WC, TyG-BMI, and C-index) demonstrated discriminatory ability for identifying the presence of diabetes, with AUC values exceeding 0.5 and all comparisons showing statistical significance (*P* < 0.05). Among these indicators, TyG-WC exhibited the strongest discriminatory performance, with an AUC of 0.735 (95% CI: 0.705–0.766), sensitivity of 73.53%, and specificity of 60.61%. TyG-WHtR ranked second, with an AUC of 0.716 (95% CI: 0.685–0.747), sensitivity of 76.83%, and specificity of 56.36%. The optimal cutoff values based on the maximum Youden index were 797.89 for TyG-WC and 5.08 for TyG-WHtR. Detailed results are presented in Table 4 and Figure 3.

**Table 4:** Comparison of diagnostic performance of obesity- and lipid-related indicators for identifying diabetes.

**Indicators**	**AUC**	**95% *CI***	***P*-value**	**Sensitivity (%)**	**Specificity (%)**	**Youden index**	**Cut-off value**
CVAI	0.640	(0.607–0.674)	<0.001	73.21	49.09	0.223	116.48
TyG-WHtR	0.716	(0.685–0.747)	<0.001	76.83	56.36	0.332	5.08
TyG-WC	0.735	(0.705–0.766)	<0.001	73.53	60.61	0.341	797.89
TyG-BMI	0.686	(0.655–0.718)	<0.001	63.55	64.85	0.284	221.98
C-index	0.585	(0.551–0.619)	<0.001	45.88	67.88	0.138	1.24

**Figure 4:**
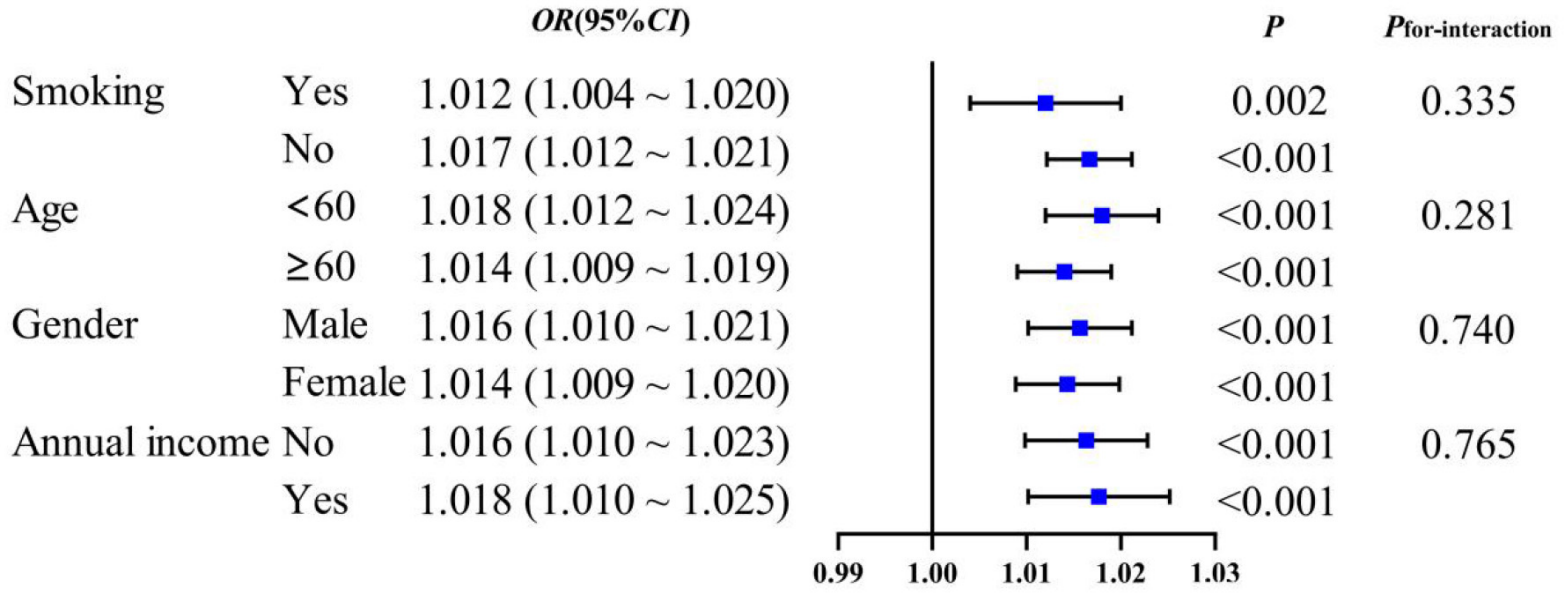
CVAI and subgroup analysis of the presence of diabetes in high-risk populations for cardiovascular disease.

**Figure 5:**
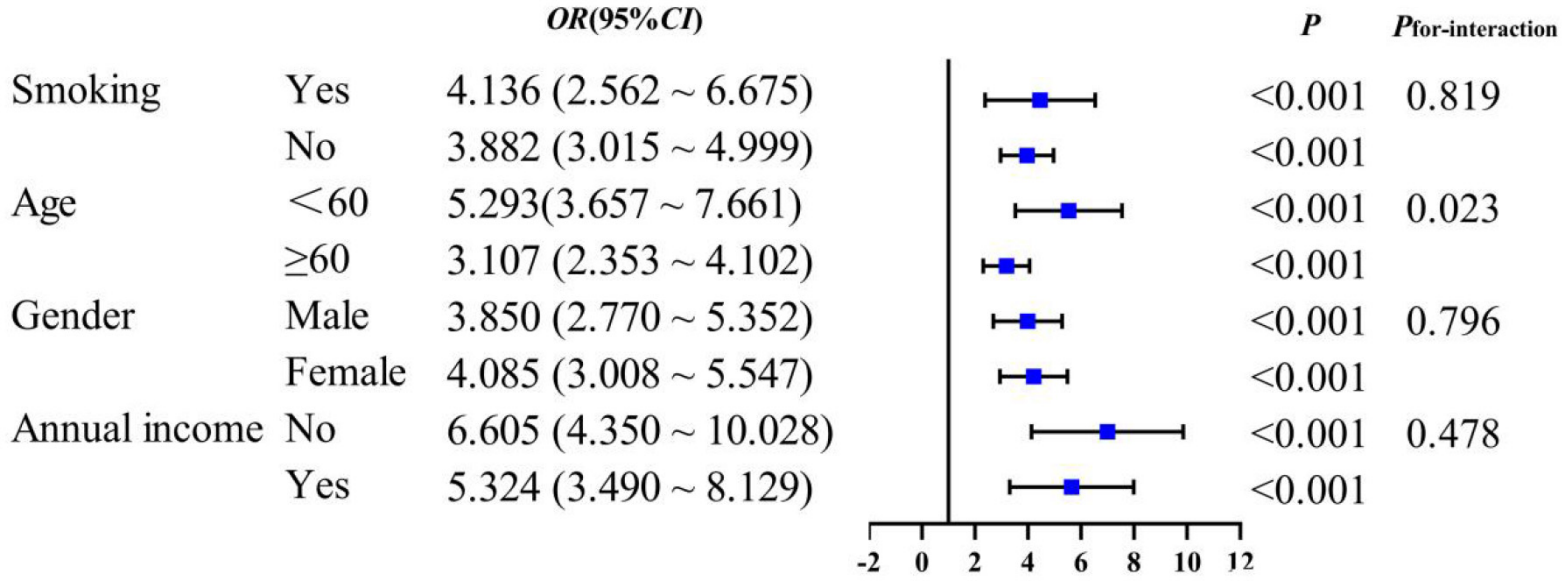
TyG-WHtR and subgroup analysis of the presence of diabetes in high-risk populations for cardiovascular disease.

**Figure 6:**
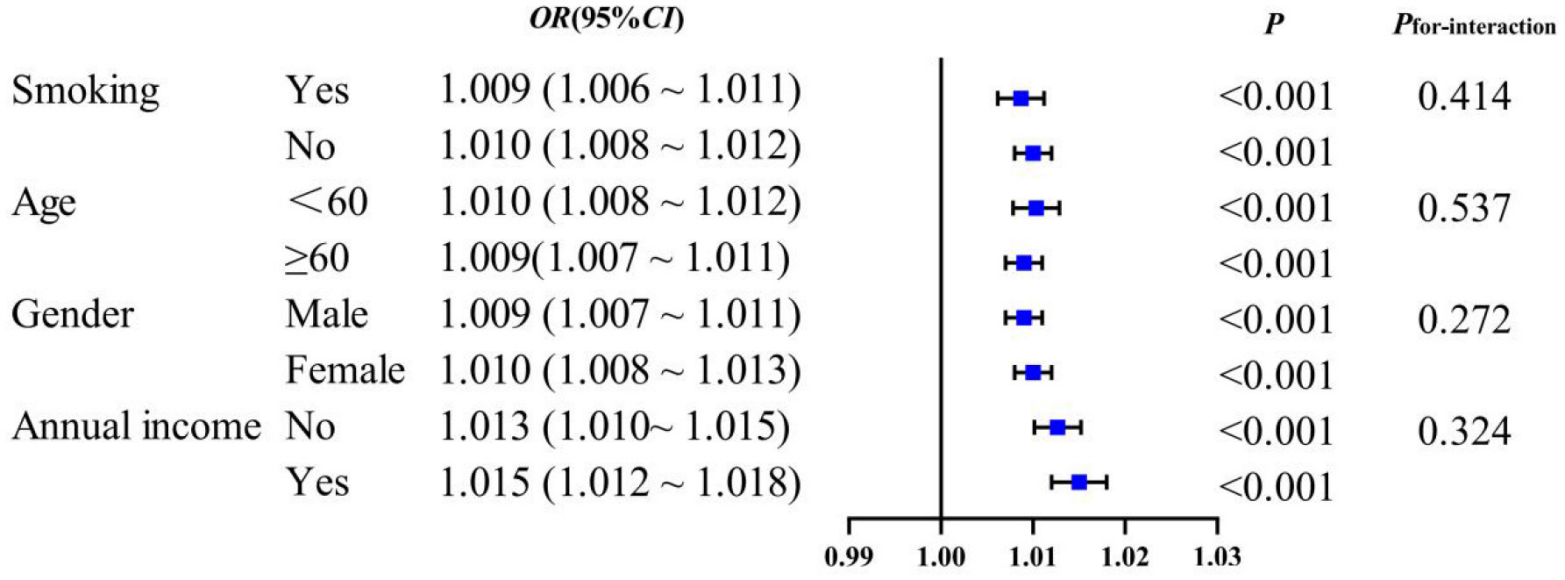
TyG-WC and subgroup analysis of the presence of diabetes in high-risk populations for cardiovascular disease.

**Figure 7:**
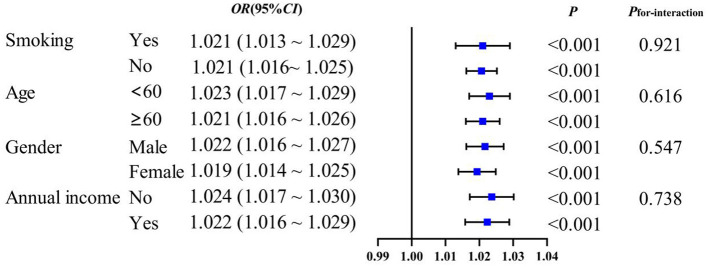
TyG-BMI and subgroup analysis of the presence of diabetes in high-risk populations for cardiovascular disease.

Subgroup analysis to evaluate whether the associations between novel obesity–lipid indicators and the presence of diabetes were consistent across different strata in the high-risk cardiovascular population, we conducted subgroup analyses using CVAI, TyG-WHtR, TyG-WC, and TyG-BMI indicators via multivariate logistic regression across different subgroups (smoking, age, sex, and central obesity). The results showed that CVAI, TyG-WHtR, TyG-WC, and TyG-BMI remained significantly associated with the presence of diabetes across all subgroups (*P* < 0.05). With the exception of TyG-WHtR, which demonstrated a significant interaction in the age subgroup (*P*_interaction_ <0.05), no interaction effects were observed for the other indicators across the different subgroups Figures 4–7.

## 4 Discussion

Diabetes remains a major contributor to CVD morbidity and mortality worldwide, posing a growing global health and economic burden (16). Obesity induces insulin resistance through adipose tissue inflammation and impairs cardiovascular function by increasing hemodynamic stress and atherogenesis (17). Dyslipidemia, characterized by elevated levels of TC, LDL-C, and TG along with decreased HDL-C, is a principal driver of atherosclerosis (18). Obesity and dyslipidemia are closely interrelated, forming a vicious cycle that markedly elevates the overall burden of both CVD and diabetes. While a large body of evidence has examined novel obesity- and lipid-related indicators, such as the TyG index and its derivative metrics, existing studies have predominantly focused on general or community-based populations (13, 19). However, Individuals at high cardiovascular risk constitute a metabolically distinct subgroup characterized by a higher clustering of central obesity, hypertension, dyslipidemia, and chronic low-grade inflammation (20). This population predominantly comprises middle-aged and older adults, in whom age-related increases in metabolic vulnerability and the long-term accumulation of adverse lifestyle factors substantially elevate the risk of diabetes. These characteristics not only increase the overall burden of metabolic abnormalities but may also modify the associations between obesity- and lipid-related indicators and diabetes. Therefore, evaluating obesity- and lipid-related indicators in this population, particularly among middle-aged and older adults, is important for the early identification of diabetes and for targeted prevention at the public health level.

This cross-sectional study included 1,603 individuals identified as being at high risk for CVD. The crude prevalence of diabetes in this cohort was 20.59%, with a standardized prevalence of 14.15%, exceeding the national diabetes prevalence of 12.8% reported in China in 2023 and aligning with previous research findings (21). These findings suggest that diabetes is more prevalent among individuals at high risk for CVD than in the general population (22). This study observed a higher prevalence of diabetes in the high cardiovascular risk population compared to the general population, a disparity particularly pronounced among middle-aged and older adults. This can be attributed to the fact that risk factors such as hypertension, dyslipidemia, and obesity within this group are not only highly comorbid but also exhibit a synergistic effect, substantially amplifying the risk of developing diabetes (23, 24). Consequently, from a public health perspective, implementing proactive screening and integrated risk management for this specific high-risk middle-aged and older population is a critical public health strategy.

This study aims to evaluate the relationship between the TyG index combined with BMI, WC, WHtR, CVAI, and C-index and the presence of diabetes in populations at high risk for cardiovascular disease. By integrating biochemical and anthropometric information, these indicators may help characterize early metabolic abnormalities and serve as complementary tools to conventional measures such as fasting plasma glucose or HbA1c. The TyG index is a widely used surrogate marker of insulin resistance and reflects both lipid and glucose metabolism, making it closely related to metabolic disturbances. Previous studies have reported notable differences in TyG-related measures between individuals with and without diabetes. For example, individuals with diabetes showed markedly higher TyG-WHtR levels, corresponding to an ~2.7-fold difference reported in earlier analyses (25). In addition, increases in the TyG index have been associated with unfavorable metabolic profiles and demonstrated moderate discriminatory ability (AUC = 0.60), outperforming single obesity- or lipid-related indicators (26). Other studies have also highlighted the utility of TyG-WC and TyG-WHtR in distinguishing individuals with diabetes (27). Taken together, these findings underscore the potential of TyG-based composite indicators to capture multiple dimensions of metabolic health, particularly in high-risk populations.

CVAI showed a significant positive association with the presence of diabetes in individuals at high cardiovascular risk (28). This finding is consistent with previous studies indicating that CVAI better reflects visceral adiposity and its metabolic consequences than traditional anthropometric indicators. Our results further reinforce its utility in characterizing metabolic abnormalities within this high-risk population. The TyG index, a validated surrogate for insulin resistance, has been consistently associated with various metabolic disorders (29). In this study, TyG-WC showed the strongest association with the presence of diabetes, suggesting its potential utility for identifying individuals with adverse metabolic profiles in high-risk populations. Restricted cubic spline analysis revealed a clear nonlinear relationship between TyG-WC and diabetes, consistent with the findings of Li et al. The prevalence of diabetes increased sharply once TyG-WC exceeded certain threshold values (30). This nonlinear pattern may reflect the point at which the combined burden of triglyceride–glucose dysregulation and central adiposity surpasses the body's compensatory capacity. At relatively low levels of TyG-WC, preserved β-cell function, adipose tissue expandability, and residual insulin sensitivity may still buffer metabolic stress, resulting in a flatter portion of the curve (31). However, when visceral fat accumulation and TG–glucose abnormalities exceed a critical threshold, multiple metabolic processes—such as increased free fatty acid flux, hepatic metabolic overload, and chronic low-grade inflammation—may deteriorate concurrently. These disturbances can substantially intensify insulin resistance and impair glucose homeostasis, producing the steeper increase in diabetes prevalence observed at higher TyG-WC levels (32, 33). further confirming the robustness and clinical relevance of this indicator. In contrast, the C-index showed limited discriminatory capacity in our cohort, differing from the findings of Ma Hongmei (34) and Liu et al. (35). This discrepancy may be attributed to differences in population characteristics, highlighting the importance of context-specific evaluation when applying adiposity-related indicators across diverse populations.

Subgroup analyses demonstrated that CVAI, TyG-WHtR, TyG-WC, and TyG-BMI were significantly associated with the presence of diabetes. Notably, an interaction between age and TyG-WHtR was observed: in individuals under 60 years of age, the association between TyG-WHtR and the presence of diabetes was more pronounced. This pattern may reflect differences in metabolic responsiveness, as younger individuals tend to have relatively preserved β-cell function, making metabolic indicators more sensitive to lifestyle-related influences such as high-calorie diets and low physical activity levels. These behaviors can contribute to insulin resistance and increased abdominal adiposity, thereby elevating TyG-WHtR levels. This finding suggests that TyG-WHtR may possess greater relevance in characterizing metabolic abnormalities among younger populations (36). Therefore, the results highlight the necessity of early screening and the implementation of targeted lifestyle interventions in younger individuals to mitigate the long-term burden of diabetes.

In individuals at high cardiovascular risk, recognizing diabetes at an early stage is important because multiple metabolic abnormalities often coexist and may exacerbate cardiovascular burden. Our study shows that TyG-WC, TyG-WHtR, and TyG-BMI—derived from combining insulin-resistance–related metabolic information with anthropometric measures—demonstrate good discriminatory ability for identifying diabetes. These composite indicators can be readily incorporated into routine clinical assessments, as waist circumference, BMI, fasting plasma glucose, and triglyceride levels are already standard components of evaluations in populations at high cardiovascular risk, which substantially overlap with aging populations worldwide. TyG-related indices can be automatically calculated within electronic medical record systems to support the early identification of metabolic abnormalities and to prompt further diagnostic testing, such as HbA1c or oral glucose tolerance testing (OGTT), when elevated values are detected. In the context of global population aging, these simple and low-cost indicators have important public health implications for large-scale diabetes screening and risk stratification among middle-aged and older adults, particularly in community and primary-care settings with limited screening resources (5). Incorporating TyG-WC, TyG-WHtR, and TyG-BMI into existing cardiovascular risk assessment frameworks may complement traditional obesity measures and support population-level diabetes prevention strategies, thereby helping to alleviate the growing diabetes burden in aging societies.

This study has several strengths. First, it focused on a unique population—individuals at high cardiovascular risk, who represent a group with substantial metabolic burden and a higher likelihood of early metabolic abnormalities. Investigating this population offers clinically meaningful insights compared with studies based on general community samples. Second, the study simultaneously evaluated multiple novel obesity- and lipid-related indicators, including CVAI, TyG-WHtR, TyG-WC, TyG-BMI, and the C-index, allowing a comprehensive comparison of their discriminatory ability for identifying the presence of diabetes. The inclusion of these emerging composite indicators provides a more integrated understanding of metabolic disturbances in high-risk individuals.

Nevertheless, several limitations should be acknowledged. Although a range of demographic, lifestyle, and clinical covariates were adjusted for, not all potential confounding factors could be fully accounted for, and residual confounding may persist. For example, factors such as sleep quality, psychological stress, and other psychosocial variables were not available in the current dataset and could influence the observed associations. Additionally, due to its cross-sectional study design, this study can only describe associations rather than infer causal relationships between these indicators and diabetes. Longitudinal studies are needed to further examine temporal changes in these indicators and clarify their roles in the development of diabetes.

## Data Availability

(1) The data analyzed in this study is subject to the following licenses/restrictions: the data supporting the findings of this study are available from the CDC of Hainan Province, though access is restricted. These data were used under license for this study and are not publicly available. However, they can be obtained from the corresponding authors upon reasonable request and with permission from the CDC of Hainan Province. (2) The data utilized in this study were derived from the “Major Public Health Service Project of the National Health Commission: Early Screening and Comprehensive Intervention Project for High-Risk Populations of Cardiovascular Diseases.” Prior to 2019, the project number was Z135080000022; after 2019, it was Z195110010004. No additional funding was generated for this experiment beyond the national project funding. Requests to access these datasets should be directed to Xue Zhou snowweek@hotmail.com.
